# Harmonization of community health worker programs for HIV: A four-country qualitative study in Southern Africa

**DOI:** 10.1371/journal.pmed.1002374

**Published:** 2017-08-08

**Authors:** Jan-Walter De Neve, Henri Garrison-Desany, Kathryn G. Andrews, Nour Sharara, Chantelle Boudreaux, Roopan Gill, Pascal Geldsetzer, Maria Vaikath, Till Bärnighausen, Thomas J. Bossert

**Affiliations:** 1 Institute of Public Health, Heidelberg University, Heidelberg, Germany; 2 Department of Global Health and Population, Harvard T.H. Chan School of Public Health, Boston, Massachusetts, United States of America; 3 Harvard University, Cambridge, Massachusetts, United States of America; 4 Dana–Farber Cancer Institute, Boston, Massachusetts, United States of America; 5 University of British Columbia, BC Women's Hospital, Vancouver, British Columbia, Canada; 6 Africa Health Research Institute, Mtubatuba, KwaZulu-Natal, South Africa; Massachusetts General Hospital, UNITED STATES

## Abstract

**Background:**

Community health worker (CHW) programs are believed to be poorly coordinated, poorly integrated into national health systems, and lacking long-term support. Duplication of services, fragmentation, and resource limitations may have impeded the potential impact of CHWs for achieving HIV goals. This study assesses mediators of a more harmonized approach to implementing large-scale CHW programs for HIV in the context of complex health systems and multiple donors.

**Methods and findings:**

We undertook four country case studies in Lesotho, Mozambique, South Africa, and Swaziland between August 2015 and May 2016. We conducted 60 semistructured interviews with donors, government officials, and expert observers involved in CHW programs delivering HIV services. Interviews were triangulated with published literature, country reports, national health plans, and policies. Data were analyzed based on 3 priority areas of harmonization (coordination, integration, and sustainability) and 5 components of a conceptual framework (the health issue, intervention, stakeholders, health system, and context) to assess facilitators and barriers to harmonization of CHW programs. CHWs supporting HIV programs were found to be highly fragmented and poorly integrated into national health systems. Stakeholders generally supported increasing harmonization, although they recognized several challenges and disadvantages to harmonization. Key facilitators to harmonization included (i) a large existing national CHW program and recognition of nongovernmental CHW programs, (ii) use of common incentives and training processes for CHWs, (iii) existence of an organizational structure dedicated to community health initiatives, and (iv) involvement of community leaders in decision-making. Key barriers included a wide range of stakeholders and lack of ownership and accountability of non-governmental CHW programs. Limitations of our study include subjectively selected case studies, our focus on decision-makers, and limited generalizability beyond the countries analyzed.

**Conclusion:**

CHW programs for HIV in Southern Africa are fragmented, poorly integrated, and lack long-term support. We provide 5 policy recommendations to harmonize CHW programs in order to strengthen and sustain the role of CHWs in HIV service delivery.

## Abstract 2

In a qualitative study, Jan-Walter De Neve and colleagues investigate how community health worker programs can be better integrated with the wider health systems in four countries in Southern Africa.

## Author summary

**Why was this study done?:**

- Community health workers (CHWs) play an important role in scaling up HIV services in the community and contribute in major ways to achieving HIV goals.
- Multiple disparate CHW programs for HIV often exist in a single country.
- The lack of coordination between CHW programs and the lack of integration of CHW programs into larger health systems may have impeded the full realization of the potential impact of CHWs in HIV.

**What did the researchers do and find?:**

- A total of 60 interviews with government officials, expert observers, and donors were conducted to investigate the harmonization of CHW programs for HIV in Lesotho, Mozambique, South Africa, and Swaziland.
- We provide key policy recommendations targeted to policy-makers and program implementers at the national, regional, and global level to strengthen and sustain the role of CHWs in HIV service delivery.
- To increase harmonization, decision-makers in HIV-endemic settings should further develop government CHW programs; officially recognize nongovernmental CHW programs; standardize CHW training, incentives, and services; provide an organizational structure dedicated to community health initiatives; involve the community in decision-making; and provide adequate and long-term resources for CHW programs for HIV.

**What do these findings mean?:**

- A range of actions are available for a more harmonized approach to CHW programs for HIV in the context of complex health systems and multiple donors.
- Increased coordination, integration, and sustainability of CHW programs for HIV may increase the impact of CHW-led HIV services and improve the health, economic, and social outcomes of their clients.

## Introduction

The importance of community health workers (CHWs) and their contribution to healthcare and health promotion have garnered increasing attention from governments, donors, health systems researchers, and planners [1]. CHWs have been suggested to play a “transformative” role in scaling up HIV services in communities for achieving HIV goals and improve linkages between those who need care and those who can provide it [2–7]. Two recent reviews of CHWs in HIV care described positive effects on HIV service organization, delivery [8], and cost [9], but highlighted the need for programs to be integrated into the wider health system for sustainability [10,11].

CHW programs are believed to be weak in many countries, poorly coordinated with fragmented attempts at improvement, and, with multiple disparate CHW programs, often observed in a single country [12–14]. There have been few examples of integrating CHW programs into national health systems and human resources for health (HRH) policy and plans. This may be impeding the potential impact of CHWs in HIV [12,14]. Fragmented and vertically supported CHW programs that are not aligned with health systems may manifest in program inefficiencies, poor accountability, and poor coordination that can block effective and sustainable programming, service delivery, training, deployment, and support to CHWs [15,16]. To strengthen and sustain the role of CHWs in HIV service delivery, “harmonizing” CHW-led HIV services (defined as increasing coordination, integration, and sustainability), appears necessary to address commonly encountered challenges of integration within country HRH plans, lack of clarity around ownership of CHW programs, poor linkages with the health system that enable effective team-based care, and inadequate focus on sustained support for CHWs [15–19]. Box 1 provides an overview of the expansion of CHW programs.

**Box 1. The expansion of CHW programs**

Large scale CHW programs date back to at least the 1970s, when health administrators increasingly realized the potential of informally trained community-based workers to assist in rapid scale-up of primary healthcare systems [20,21]. During this time, CHWs were seen by many as a stopgap measure, and they often failed to receive the systematic support and formalized structure of other health system cadres [14]. Although CHWs have played an important role in primary healthcare strategies across many contexts, CHW programs were often based on immediate need to address critical health issues such as the HIV epidemic [22]. The net effects of this rapid implementation can be difficult to disentangle. While the lack of formalized structure had serious implications for program sustainability, it also allowed for experimentation. The skills, training, and objectives of CHWs varied widely across settings as individual efforts sought to innovate and respond to local needs.

As the HIV epidemic matures, the emphasis of CHW programs is transitioning to one of long-term care and activities beyond health promotion [23]. There is an increasing expectation that CHWs will participate in disease surveillance and data collection, diagnosis and referral for care, and antiretroviral therapy (ART) provision [10]. As this transition to greater responsibility for CHWs has occurred, these cadres and the broader “community health system” have received increased scrutiny [24,25]. Researchers and policy-makers have noted many challenges to implement task-shifting for HIV treatment and care, including integration of CHWs into larger health systems [10] and long-term political and financial support of CHW programs for HIV [26]. Additionally, heavy reliance on donor funding for many CHW cadres supporting HIV service delivery raises urgency for greater consideration of long-term support [19,22,27]. There is an increasing need to consider how to streamline CHW-led HIV activities [28].

In recent years, alignment of CHW initiatives has been promoted through several policy initiatives [13,29–31]. The Global Health Workforce Alliance (GHWA)—an alliance of government leaders, donors, health workers, and civil society—called in 2013 for a coherent and harmonized approach to CHW support within countries [13,32] and articulated 3 guiding principles for such harmonization (the “three ones”): one national strategy, one authority, and one monitoring and accountability framework [13]. The GHWA’s public commitment brought critical attention to the harmonization of CHW programs for HIV because of the history of CHW cadre creation specific for HIV. Harmonization has become increasingly important with expansion of CHW HIV responsibilities for achieving 90-90-90 goals [7]. In February 2017, UNAIDS announced the need to scale up the use of CHWs [33]. These initiatives suggest that disease-specific organizations are investing in scale-up of CHW programs [7,33].

Harmonization of CHW programs can occur along a number of dimensions: within the context of this study, we identify coordination among development partners, integration into the broader health system, and assurance of the program’s sustainability to be 3 priority areas. Among CHW programs, coordination efforts seek to reduce duplication, fragmentation, confusion created by competing models, and overlap of responsibilities of differently trained CHWs in the same geographic areas [13,34]. Integration refers to the absorption of CHW programs into existing networks of larger health systems, primarily Ministries of Health or a large private provider (nongovernmental organization [NGO] or commercial) [13]. Sustainability is a key issue for CHW programs supporting HIV services that have been solely supported by donor programs, such as the US President's Emergency Plan for AIDS Relief or the Global Fund [13,17,35]. S1 Box provides additional details on each of these priority areas.

Harmonization of CHW programs may have been further impeded by lack of understanding of key harmonization issues, experiences, and lessons learned to date [13]. Systematizing collective learning and understanding of CHW program harmonization is a practical and formative step towards building evidence in this field. This study contributes new evidence on the harmonization of CHW programs for HIV in two ways. First, it describes factors that help or hinder efforts toward achieving a more harmonized approach to implementing large-scale CHW programs in the context of a generalized HIV epidemic, complex health systems, and multiple donors. We identify reasons for keeping fragmentation for innovations and for exploring different approaches. Second, the study provides policy recommendations for a more harmonized approach to CHW programs for HIV. The premise of the study was not to suggest that all CHW programs should be harmonized but rather to assess mediators and trade-offs associated with harmonization.

## Methods and materials

### Ethics

This study was reviewed by the Harvard T.H. Chan School of Public Health Institutional Review Board and considered exempt from full review since it is based on anonymous data with no identifiable information about survey participants. Additionally, the study was approved by the national ethical committees of Lesotho, Mozambique, South Africa, and Swaziland.

### Design

We conducted 4 descriptive country case studies between August 2015 and May 2016. We purposively sampled 4 countries heavily affected by the HIV epidemic and that have had some success in integrating donor-supported CHW-led activities into their national health systems: Lesotho, Mozambique, South Africa, and Swaziland. HIV prevalence among adults aged 15–49 ranged from 10.5% in Mozambique to 27.7% in Swaziland [36] (see S2 Text for additional contextual information on CHW programs delivering HIV services in each country). While the selection of case studies in any comparative analysis is to some extent arbitrary, the 4 countries are geographically and historically closely related, which implied that their broad context would be relatively similar compared to other potential countries. By attempting to hold the epidemic and broad context constant among the 4 case studies [37], our aim was to focus on mediators of harmonization at the intervention, stakeholders, and health system levels, which were key elements in our analytic framework [34]. Qualitative data were collected in each country through semistructured face-to-face interviews.

Three categories of respondents were eligible to take part in the study. First, we interviewed government officials, defined as key staff involved in the programmatic and coordination roles of provincial or national CHW programs. This included officials involved in planning and coordinating activities, technical staff involved in the oversight of government-funded CHW activities for HIV, and officials participating in national advisory boards and working groups related to CHW work. Second, we interviewed donors, defined as staff involved in determining which programs to fund, those who oversee grants or loans funding CHW activities for HIV, implementation and evaluation of CHW programs providing HIV services, and those sitting on national advisory boards and working groups related to CHW work. Third, we interviewed expert observers, defined as any staff working with ongoing CHW activities for HIV at a nongovernmental (or faith-based) organization or academic expert within a participating country. From each participating organization, efforts were made to include at least 1 technical staff and 1 staff involved in planning and coordination of activities. Exclusion criteria were age less than 18 years old at the time of the study and inability to provide informed consent.

### Interviews

The interview process was single-staged. We used purposeful sampling to identify interviewees by constructing a preliminary list of 15–20 relevant stakeholders prior to arriving in each country. We chose to interview government officials, expert observers, and donors for three reasons. First, stakeholders were likely well informed about the programmatic and coordination roles of provincial and national CHW programs. We sought participants who were aware of the coordination, planning, sustainability, and operations of CHW-led activities, such as the implementation designs and operational processes of specific CHW programs. Second, we aimed to identify participants likely to interact with multiple CHW programs and who had responsibilities above and beyond the CHW program that they were primarily involved with—in order to provide insights into approaches to better coordinate programmatic activities and governance across CHW programs. In other words, we sought participants likely to have experience with existing efforts to reduce duplication, fragmentation, inefficiencies created by competing models, and overlap of responsibilities of differently trained CHWs in the same geographic areas. Finally, we were interested in assessing power structures and how participants navigated the complex web of political actors and institutions involved with coordination and harmonization of CHW programs at the provincial and national level. We assessed key drivers of political agenda setting and policy diffusion around harmonization (S1 Table: Country data analysis sheet: “political strategies for harmonization”) [38,39]. All participants were involved with national, nongovernmental, or disease-specific CHW programs that deliver HIV services including: education, testing and counseling, and antiretroviral therapy (ART) adherence support. For consistency and completeness, we wrote semistructured interview guides. Interviews lasted approximately 1 hour and aimed to identify likely facilitating and impedimentary contributors to harmonization. Interviews were conducted by data collectors with experience in qualitative data collection; either in English, in a mix of the country’s national language and English, or in the country’s national language with professional interpretation. Each interviewee was informed of the purpose of the study, our intention to take notes, and our process for handling interview data. Interviewees provided verbal consent. Interviews were recorded and transcribed into electronic text.

### Analysis

First, we identified major issues and relationships surrounding harmonization of CHW programs based on reviewed documents. We conducted a narrative review of existing published and grey literature building on recent work on coordination, integration, and sustainability of CHW programs [13,29,40]. We used an analytic framework developed ex ante to map overarching findings and inform the design of our semistructured questionnaires. Additional details on our narrative review and analytical framework are provided in S1 Text and elsewhere [41]. Second, to assess key constructs of our framework for each country, we analyzed data from interviewees. Transcripts were read in their entirety, coded to indicate whether they provided information on harmonization, and extracted data was summarized and grouped under priority areas of harmonization and components adapted from the analytic framework (S1 Table). The 3 priority areas of harmonization (coordination, integration, and sustainability) are interlinked components. We, therefore, additionally assessed how priority areas of harmonization related to each other. Outcome variables for this analysis were coordination, integration, and sustainability. Explanatory variables were mediators of harmonization of CHW programs, categorized by 5 components of our analytic framework.

## Results

We interviewed a total of 60 participants including government officials, donors, and expert observers in each of the four countries. Selected characteristics of the study participants are shown in Table 1. The country case studies proceed as follows. First, we identify key themes arising from the interviews related to each priority area of harmonization and categorize them by components of our analytic framework, separated for each country. We include country-specific examples, list success stories and failures, and provide insights into advantages and disadvantages of harmonizing of CHW programs. In Table 2, we show an assessment of the current state of harmonization of CHW programs, separated by country. Second, we list key mediators of harmonization of CHW programs identified by study participants. Finally, we compare all 4 countries and show overarching findings. In Table 3, we show overarching mediators of harmonization. In Table 4, we map overarching findings for priority areas for harmonization in our analytic framework.

**Table 1:** Selected characteristics of the study participants.

	Lesotho	Mozambique	South Africa	Swaziland	Total
Number of participants	13	15	16	16	60
Expert observers	8	6	7	9	30
Government officials	3	3	5	5	16
Donors	2	6	4	2	14
Range of positions					
Country director/chief-of-party	✓	✓	✓	✓	✓
Deputy (country) director	✓	-	✓	-	✓
Head of department	✓	✓	✓	✓	✓
Program director/head	-	✓	-	✓	✓
Program (assistant) manager	✓	✓	✓	✓	✓
Program specialist	-	✓	-	✓	✓
Researcher	-	✓	✓	-	✓
Expert/consultant	-	✓	✓	✓	✓
Provincial liaison	-	-	✓	-	✓
Gender					
Female (%)	8 (62)	7 (47)	10 (63)	11 (69)	36 (60)

*Notes*: Positions included the following: head of department; head of cluster; independent ministry of health (MOH) consultant; monitoring and evaluation manager; orphans and vulnerable children/prevention manager; health systems researcher; community oriented primary care expert; deputy medical coordinator; community linkages program officer; community systems advisor; prevention of mother-to-child transmission (PMTCT) coordinator; and infectious diseases specialist. Community health worker (CHW)-led HIV services covered HIV education, door-to-door HIV testing campaigns, pre- and post-test and antiretroviral (ARV) treatment adherence counselling, monitoring ARV adherence, PMTCT, home-based care delivery, and the community supply of ARVs.

**Table 2:** Assessment of current status of harmonization by study participants.

*Area of harmonization*	Lesotho	Mozambique	South Africa	Swaziland
*Coordination*	No national commission currently to oversee all the different CHW programs; no centralized mapping available of partners; national versus district-level coordination	National versus province-level coordination; parallel CHW services; CHWs are overburdened; lack of consistent meetings among partners to coordinate	Duplication of services; different meetings that groups are able to come to, however it is mostly voluntary; lack of trust in government	Many stakeholders and structures; lack of consultation between CHW programs; poor coordination between health priorities; stipends for CHWs versus volunteerism
*Integration*	Unlikely that government will be able to integrate all CHW programs into the MOH; only 1 cadre currently recognized by the MOH (VHWs)	Wide range of donors across provinces; provincial vis-à-vis national integration; unlikely that government will be able to integrate all CHW programs into the MOH	Ward-based outreach teams that are integrated in clinics; clinic committees; differences between provincial health systems; provincial vis-à-vis national integration	Only 1 cadre currently recognized by the MOH (RHMs); lack of standardized training or stipend; multiple government stakeholders
*Sustainability*	Short-term projects that address niche needs; no clear long-term plans and professional development for CHWs; different career paths for CHWs	No clear long-term plans for CHW programs; NGOs working out of grants with limited plans for sustaining funding; government is unlikely to be able to sustain CHW funding without external support	NGOs provide long term services but under limited government purview; NGOs working out of grants without plans for sustaining funding	Underfunded CHWs; government focuses on RHMs; funding through donors is insufficiently coordinated through MOH; awareness of need for long-term plans; CHW attrition

*Notes*: Table 2 represents a summary of views of study participants and is not intended to be a systematic review of the status of harmonization in each country. CHW, community health worker; MOH, Ministry of Health; NGO, nongovernmental organization; RHM, rural health motivator; VHW, village health worker

**Table 3:** Mediators of harmonization mentioned by study participants.

*Mediator*	Lesotho	Mozambique	South Africa	Swaziland	Overarching theme
*Facilitators*					
Large national CHW program	✓	✓	✓	✓	Yes
National government structure dedicated to CHWs	✓	✓	**-**	✓	No
Recognition of non-MOH CHW programs	✓	✓	✓	✓	Yes
Clear definitions of CHWs, CHW program	✓	**-**	**-**	✓	No
Similar job descriptions, matched pay rates	✓	✓	✓	✓	Yes
Broad community healthcare package	✓	**-**	✓	✓	No
Engagement of community leaders	✓	✓	✓	✓	Yes
CHWs linked to health facility	✓	**-**	✓	✓	No
National data collection	**-**	**-**	✓	✓	No
National training	✓	✓	✓	**-**	No
Political support for harmonization	✓	✓	✓	✓	Yes
*Barriers*					
CHWs focused on HIV alone	✓	**-**	**-**	✓	No
Multiple ministries with different guidelines	✓	**-**	**-**	✓	No
Variation by province/district	✓	✓	✓	**-**	No
Lack of continuing education/career path	✓	✓	**-**	✓	No
Lack of accountability of non-MOH programs	✓	✓	✓	✓	Yes
Paper-based measures	**-**	**-**	**-**	✓	No
Lack of funding	✓	✓	✓	✓	Yes
Lack of human resources	✓	✓	✓	✓	Yes
Lack of technical resources	✓	✓	✓	**-**	No
Political turnovers and instability	✓	**-**	✓	**-**	No

*Notes*: Table 3 represents a summary of views of study participants and is not intended to be a systematic review of mediators of harmonization in each country. CHW, community health worker; MOH, Ministry of Health

**Table 4:** Mapping harmonization findings to the analytic framework.

*Framework*	Coordination	Integration	Sustainability
*Problem*	Coordination with other health priorities beyond HIV; a standardized community healthcare package, including TB, MNCH, nutrition, personal hygiene	Movement toward more holistic care; variability in health priorities between national and subnational levels	National CHWs seem overall the furthest reaching and carry many skills; limited geographical coverage of HIV services
*Intervention*	Fragmented programs in addition to national CHWs; available cadres with specialized skills are difficult to manage and measure; parallel training and structures	No equivalence between national CHWs and many other CHW types; variation in training within CHWs and with other health workers; piecemeal supervision	Wide range of CHW responsibilities; lack of harmonized incentives, continuing education, and career prospects; low morale; flexibility of CHW programs
*Stakeholders*	Wide range of stakeholders; regular meetings among stakeholders to coordinate, but often “after the fact” and limited commitment to these meetings	Involvement of multiple ministries without clear path of integration into a single ministry; many “free-floating” CHW programs; high dependence on external actors and resources	Community buy-in to sustain CHW programs, navigate local politics, and to continue to identify community members that could serve as CHWs
*Health system*	Lack of organizational structure specifically dedicated to CHWs; coordination with local health and training facilities; ward-based outreach teams	Recognition of national CHW program only; inability of MOH to absorb fragmented programs; geographical variation by provinces and districts; parallel supply chains	Inability to fund and support many necessary cadres long-term; limited training refreshers for CHWs; CHW attrition; limited human and technical resources
*Broad context*	Strong political support across government levels and stakeholders for community health initiatives; geography of country	Need for program compatibility with local structures; CHW and country demographics; educational attainment and literacy rate	Alignment with community norms and needs; community engagement; political and economic stability; high support from external actors

CHW, community health worker; MNCH, maternal, newborn, and child health; MOH, Ministry of Health; TB, tuberculosis.

### Case study: Swaziland

#### Swaziland: CHW programs for HIV

Swaziland has a long-standing national CHW program, the “Rural Health Motivators” (RHMs). In addition, numerous donors and NGOs have created many CHW programs that deliver services including HIV, such as the “expert clients” and “mothers2mothers.” Study participants mentioned that newer donor-supported CHW programs have attempted to go through the RHM system to utilize RHMs for service delivery, but the RHM system may already be overburdened with wide-ranging existing responsibilities beyond HIV services. Other programs circumvented the RHM system by establishing their own CHW program, but without equivalency in the Ministry of Health (MOH). This has led to a parallel system largely accountable to whomever funds them.

"*I don’t think we have any well-coordinated system at the moment from a government point of view that is supporting the community health work being done. …I think currently it is more ad hoc, you know*."

(Government official)

While the RHMs are the furthest reaching and have many skills, parallel programs with specialized skills were difficult to manage and assess by the MOH. Groups that did not work via the RHMs were able to set their own training with what they think is necessary outside of major oversight. Interviewees mentioned a lack of a standardized system of levels in training. As a result, if the non-RHM programs were to be integrated into the MOH, it was unclear whether they would be accepted “as-is” or would need to go through RHM training because their training is either incompatible with RHM responsibilities or outdated. Similarly, there was no harmonized system of incentives across RHM and donor/NGO CHW programs, an issue which, according to respondents, affected morale and buy-in of CHWs. Respondents mentioned that CHWs in non-RHM programs generally received higher compensation from donors or NGOs.

Because most grants and funding cycles were on a similar timeline, predictable schedules gave many CHW programs time to seek alternative support in case their current funding ceased. However, it seems like few had and, if funding ceased, there was not a strong sense among study participants that the MOH would consider the non-RHM programs indispensable. The MOH seemed largely focused on the RHMs, leading to fewer resources to absorb other programs.

Participants also brought up trade-offs associated with harmonization of CHW programs. It was not clear that the solution to difficulties with fragmented CHW programs lay in integrating them entirely into a national health system or large private provider.

“*A disadvantage [to harmonization] could be that people feel that they are being restricted…as opposed to them exploring other fields of providing support*…”

(Donor)

#### Swaziland: Stakeholders, health system, and broad context

Study participants described difficulty coordinating CHW activities across the MOH and the Ministry of Tinkhundla Administration and Development, which oversees smaller administrative units (called Tinkhundla). CHWs seemed a joint effort between the two ministries, which complicates chain of command and accountability. Participants also highlighted the need to involve community leaders for sustainable solutions. Multiple interviewees worried there would be community distrust without enhanced local involvement. While national-level/district-level leaders were important for top-down integration, community buy-in was necessary to keep programs going and to continue to identify community members that could serve as CHWs.

While the various stakeholders involved with CHW activities did appear to attend meetings from time to time to coordinate among themselves, there was no system requiring them to attend. These meetings occurred only after NGOs or CHW programs were established within their district or health services area, as opposed to being timed to coordinate initial implementation and planning. Wide-scale integration of CHW activities was never attempted and capacity would need to be built within the MOH itself. Supervision of CHW programs, for instance, was still largely piecemeal according to study participants. Interviewees also brought up concerns of differences of educational level between CHWs—and high attrition among CHWs, particularly among younger CHWs.

#### Swaziland: Facilitators of harmonization

A key facilitator was the government’s national RHM program, which contributes to centralizing and standardizing procedures. The government aimed toward greater standardization and this has materialized in, for instance, increased data collection by NGOs and attempts to increase oversight of CHW programs. Interviewees mentioned success stories initiated by the government to increase harmonization, including integration of specific CHW programs into the RHM program and creation of a basic community healthcare package (including essential CHW services) to facilitate integration and standardization. Red Cross volunteers, for instance, were integrated into the national CHW program.

“…*in the past 3 years the Ministry has really tried its best to harmonize that, for example like there are many things, Red Cross volunteers, we upgraded into the RHM program, that was one improvement to strengthen the community based systems*."

(Expert observer)

The cause of CHW-led HIV services also had strong political support at all levels of government. Other facilitators of harmonization included similar job descriptions and matched pay rates from the MOH, leading to less competition between non-MOH programs and the national RHM program, and coordination through health facilities by the RHMs.

#### Swaziland: Barriers to harmonization

A key barrier reported by respondents was the lack of formal recognition of non-RHM programs. NGOs each hired their own CHWs who are not recognized by the government (e.g., “mentor mothers” and “adherence counselors”). Respondents mentioned that it was unclear to them what their equivalents would be in the MOH or if one could parse the variety of training levels into a coherent system. There were many free-floating CHWs unaffiliated with a centralized CHW agency and/or not linked to a health facility.

“…*of course I would love to, for them to be handed over to the MOH. But if the MOH does not recognize a cadre as a cadre, then how are we going to do that*?”

(Expert observer)

Another major barrier was poor commitment to interagency meetings to coordinate CHW programs. Despite regular meetings between stakeholders, some organizations preferred not to attend or directly competed with each other, leading to poor coverage of services. Respondents also voiced concerns about the lack of continuing education or possibilities for career advancement for CHWs. Younger CHWs were difficult to retain in the program when incentives are small or nonexistent and career prospects are limited. Other barriers included the use of paper-based management systems and difficulty summoning RHMs for refresher trainings when in the field. This made updating their programs and information more challenging.

### Case study: South Africa

#### South Africa: CHW programs for HIV

Interviewees mentioned that many NGO-based CHWs work outside the national health system; and they were worried about limited accountability to the Department of Health (DOH), leading to duplication and/or poor coverage of services. The government had tried to exert influence over external actors coming into the area and to coordinate CHW services for HIV. However, participants were pessimistic about how effective efforts to harmonize CHW services were. Interviewees also mentioned there had been piecemeal entries of NGO programs into the national system, which avoided the need for the national system to absorb many CHW programs at once with limited capacity to support all of them. Some NGOs have handed specific programs to the DOH or were working to strengthen current DOH programs to provide better worker benefits or training for the DOH. Participants suggested that important variability in CHW harmonization remained among the various South African provinces. The Gauteng province, for instance, has integrated CHW programs into the government program and were running CHWs through their DOH. Therefore, integration efforts may be regional rather than nationally-based.

"*In the Limpopo province they have a strong linkage with the NGOs, in fact the CHWs are run by the NGOs… In Gauteng…the DOH decided to depart ways with the NGOs…they were talking about integration of CHWs from NGOs to the national DOH… Those that got funding continue because they don’t rely on the national DOH fund, they continue with the CHWs. Then the others were taken up by the Gauteng national DOH*…"

(Expert observer)

For NGO-based CHW services working entirely from grants, few had plans for continuation after the current funding ended. Participants worried that the DOH did not have sufficient funds to financially support these programs. Although NGO- and government-based programs continued to apply for grants and receive funds, this also resulted in less government purview.

#### South Africa: Stakeholders, health system, and broad context

Participants mentioned a wide range of stakeholders involved with CHW programs for HIV. Most coordination of CHW-led HIV services occurred at the regional level vis-à-vis the national level. Some NGOs worked with district coordinators, while others worked with provincial coordinators that overrode districts. While there was broad, lower-level political support for harmonization of CHW programs, respondents expressed the need for increased top-down measures from the national level, as opposed to (merely) grassroots bottom-up directives.

"…*that is where the biggest failure is, at the top*."

(Expert observer)

According to study participants, the health system’s primary care program was “reengineered” towards an all-encompassing primary and preventative format rather than focused on HIV alone; aiming to try and reign NGOs and other disparate programming back in and provide baseline care with more preventative measures that informants felt would be more sustainable long-term. CHW programs were central in this restructuring process, and participants suggested that there is long-term investment and interest in holistic community health initiatives and CHW services. There was need for additional resources, however, as the lack of human and technical support for CHWs was at the forefront in many stakeholders' minds. Respondents were concerned that current resources are insufficient to meet CHW programs needs for HIV if integrated into the national system.

"*So for me the issue is not so much for HIV programming, it’s not so much about government. All of these are symptoms of a weaker health system that is unable to respond. It’s unable to respond because we are not investing more resources into producing more nurses, more doctors, and other health professionals and other players to support*."

(Government official)

#### South Africa: Facilitators of harmonization

Participants mentioned that use of ward-based outreach teams and health facilities made it easier to coordinate different CHW programs. Working through health clinics was a way of introducing households into the departmental health system. Ward-based outreach teams were also able to address other health issues in addition to HIV, such as nutrition, personal hygiene, and sanitation. Another key facilitator was use of a harmonized training system for CHWs, which facilitates equivalencies between various CHW programs. According to many participants, the most successful incentives were increased career advancement and support structures. Similarly, use of common supply chains by CHW programs made it easier to track what materials were being disseminated into the community and to create and reach targets. Using similar operational processes made it easier to map activities and eventually integrate CHW programs into the government structure. Other facilitators mentioned by participants included development of the national CHW program, piecemeal integration of NGO-based programs into the health system, a national electronic data collection system, and community engagement to navigate local politics and rivalries between chiefdoms.

#### South Africa: Barriers to harmonization

Interviewees worried that involvement of multiple stakeholders at various government levels led to different standards being implemented. It appeared difficult to accomplish (full) harmonization based on strong discrepancies among CHW programs, including among regional governments themselves, and their abilities to integrate various CHW programs delivering HIV services. Participants placed much emphasis on the role of regional government in harmonizing CHW programs. Other barriers included NGO-based CHW programs working outside the current health system, a lack of planning for the future given short-term funding such as the possibility of transitioning to governmental ownership and frequent political turnovers, in addition to limited human, financial, and technical resources.

“…*You know in South Africa when a new politician comes he changes everything and there is no continuity. So he comes with his own dream and says this is my dream now this is how we work things*."

(Expert observer)

### Case study: Lesotho

#### Lesotho: CHW programs for HIV

“Village Health Workers” (VHWs) is the main program in Lesotho and operates through the MOH. Participants mentioned that only one program was recognized by the government, making it difficult to obtain a complete picture of all CHW programs operating in Lesotho—and what CHW services are being provided and where. Other CHW programs involved with HIV services include the “mentor mothers” and “lay counselors,” generally supported by NGOs and/or donors. Participants mentioned that external actors supported a wide range of lay workers for various specific tasks (either ad-hoc or their entire programmatic focus). Many respondents expressed concern over other partners working in Lesotho for short-term projects that addressed niche needs, but with no sustainable solution to community issues. There was a sentiment that many simply left at will without long-term plans to integrate into existing networks of larger health systems, such as the MOH or large private providers.

“…*You have huge numbers of donors…all of them have implementing partners, many of whom have community-based activities. All the donors, have their own priorities*."

(Expert observer)

There appeared to have been efforts to centralize and standardize training of CHWs, such as through development of a memorandum of understanding (MOU) between the MOH, donors, and NGOs. However, this was a difficult process, involving multiple partners and priorities. Given the low-resource setting, it has been challenging to require external actors to sign the MOU or submit regular progress reports to the MOH. Sustainability of funding was a major concern. There was little confidence among participants that the government was capable of integrating all CHW programs into the MOH.

Conversely, respondents brought up potential disadvantages to the harmonization of CHW programs. An appealing component of CHWs was their ability to function in nonhomogenous ways. CHWs were also uniquely positioned to know the important issues affecting the communities in which they live and work. Respondents were concerned with losing flexibility to tailor services to community needs and thought a stronger central government might misunderstand community needs and impose inappropriate care.

“*So there might be some people who might be laid off within the [harmonized] structure… Because sometimes we may find even for [HIV] testing, we need people who have good eyesight and unfortunately there are some trusted within the communities where there are certain things they cannot fit the criteria, which is a pity really*.”

(Expert observer)

#### Lesotho: Stakeholders, health system, and broad context

Respondents referenced a wide range of high level stakeholders within the government itself (including the royal family of Lesotho), in addition to a range of independent partners each with their own health initiatives. Government services were coordinated through District Health Management Teams (DHMTs), unlike those from NGOs or community-based health organizations. DHMTs operate fairly autonomously, with a variety of services that are not required to report to the MOH. According to participants, the MOH lacked a clear sense of what each actor was doing and at which level of government. On the other hand, the DHMTs enabled some structural capacity to support district-specific community. However, at the time of interviews, there was no single entity in charge of CHWs at the national level, and individual districts determined how they were coordinated. NGOs followed the MOH policies mostly voluntarily, because the MOH did not mandate it.

"…*We do not have a sort of standardized way which is defined that we will meet every so often, but we do meet. There are meetings…where we actually talk about the VHW program, what they are doing, and so on*."

(Government official)

Participants also mentioned a lack of balanced demographics among CHWs (few male CHWs) and the complex dynamics surrounding trust and stigma in the close-knit societies in Lesotho, where nearly everyone knows their neighbors well. These broad demographic and cultural concerns appeared to affect delivery of HIV services provided by CHWs. Other broad contextual factors included the relatively small size of the country, which appeared helpful for reaching all populations effectively (although it, conversely, limited the country’s financial and physical resources), mountainous geography of the country, and high rates of immigration.

#### Lesotho: Facilitators of harmonization

Because there is one relatively well-defined CHW program currently under the government's purview, there was some standardization for CHW services offered through the government, regardless of NGO activities. By having community-based organizations working through the government and its health system, there was already a system in place that could be further developed to improve future integration. Additionally, there are a variety of NGOs that either use CHWs and/or a community health center, as opposed to conscripting their own workers, which helps strengthen the country’s CHW program further. Participants also highlighted that, despite strong political influences, most stakeholders seemed committed to greater nonpartisan efforts and working across departments. According to participants, the Prime Minister of Lesotho, for instance, hoped to prioritize CHWs in the future; and many said that the MOH was trying to increasingly focus on community health programs. Other important facilitators of harmonization of CHW programs that were mentioned by participants included the use of local health facilities by CHWs (leading to increased accountability), the existence of technical working groups created by the government, and added governmental supervision. These were instrumental in bringing together multiple stakeholders to coordinate CHW activities.

"*The reason why I think [our ART program] was a success was because from the district hospital down to the health center and the community council there was accountability*."

(Expert observer)

#### Lesotho: Barriers to harmonization

According to participants, a major barrier was the lack of a national agency to oversee the various community health programs. Because there was no national governing board in charge of CHW coordination, “public health nurses” supervised the different programs of NGOs in addition to supervising their own CHWs. Many were concerned that there was no national oversight or headquarters in the MOH fully dedicated to CHW programs. A second major barrier was limited financial and organizational resources and capacity to absorb CHW programs, particularly as increasingly more tasks were shifted on the main CHW programs.

“*For a long time you had only one person responsible for community health worker programs and…expected to do this on a national basis, that’s impossible*.”

(Expert observer)

Some participants mentioned a need for a standardized training program. Some partners providing services were in charge of coordinating their own training programs, which may or may not compare well with one another. There was also little standardization among incentives provided by the various CHW programs, possibly leading to frustration. Participants mentioned that the general lack of funding and differences in incentives (CHWs working for the government, in particular, were paid very little) led to dissatisfaction among CHWs and CHW attrition in their programs. Other barriers to harmonization included a lack of consolidation of supply chains (e.g., external actors would supply their own kits to CHWs as necessary), difficulty reaching vulnerable populations—such as “herd boys” who tend cattle and sheep, and sex workers, and the existence of multiple political actors with various community health initiatives and agendas, which made navigating the political system more difficult.

“…*that somebody will be paying them while other, those that are working for government probably are not paid or paid little amount of money. It brings dissatisfaction*.”

(Expert observer)

### Case study: Mozambique

#### Mozambique: CHW programs for HIV

Mozambique’s national CHW program is known as the “Agentes Polivalentes Elementares” (APEs) (essential [or elementary] multipurpose agents). While initially focused on HIV and home-based care, most CHWs increasingly cover other health priorities. Because the CHW program covers a broad range of activities, many external actors appeared to work on the training and supply side in order to bolster the APEs (as opposed to creating their own program). The multitude of responsibilities, however, possibly reduced the availability of CHWs to complete additional tasks when not provided with adequate supplies and human resources to support them.

"*They [CHWs] have a curative role, to treat people. It means that they are failing to do prevention and health promotion. We as a Ministry, we are failing a bit this role of the CHW, for health promotion and prevention, because we are just putting the CHW as a substitute of health care worker*."

(Government official)

Although most HIV services supported by external actors appeared to work through the national CHW program, a few bypassed them instead, and some CHW programs existed entirely outside the national program. According to participants, different actors have come up with a range of initiatives for HIV services such as HIV adherence tracing and defaulter tracing. However, even if these services are not formally organized under the government program, the government was aware of them and engaged with them to increase harmonization. Additionally, there was variation among geographical regions in donors and CHW programs. Because of the fragmented nature of the provincial programs, the government aimed to restructure current programs to provide similar services through the national chain of command/pipeline in the future.

#### Mozambique: Stakeholders, health system, and broad context

The main stakeholders were the MOH and provincial government, followed by donors, NGOs, and community leaders. Specifically, there was a small structure within the MOH designed to manage CHWs, with an independent budget, and the aim to coordinate and supervise CHW programs but not direct their implementation. Part of the current coordination effort included registration of CHWs to track the services provided, and CHW training to some extent. APE coordination meetings occurred once or twice a year, and a technical working group existed to coordinate and plan HIV services among different partners. However, stakeholders did not attend consistently. Uncertainty remained regarding the different NGOs acting within an area and the services delivered. Some partners bypassed MOH guidelines when training CHWs (such as for training about CHW-based defaulter tracing) and the delivery of CHW-led services, leading to confusion and delays with disseminating supplies and services for HIV.

Respondents identified the need for increased MOH involvement to identify solutions that could help sustain progress made long-term. Some communities became dependent on HIV services supported by donor-funded programs. However, plans for continuation of the programs if and when the donors discontinued funding were not made. In general, respondents did not believe that the government is technically and financially able to absorb the various CHW programs in the near future. Additionally, much of the planning with partners and implementation occurred at the provincial level, which created discrepancies between HIV services being delivered by different CHW programs and made it more difficult to integrate them into a single, national framework.

#### Mozambique: Facilitators of harmonization

According to participants, a major facilitator was the existence of an entity within the MOH dedicated to supervising community health initiatives. Moreover, central level actors such as the Minister of Health also appeared to see merit in the CHW program and publicly supports it, which led to broad political support. However, participants noted that this has not translated into significant allocation of resources. Participants also mentioned that there was a clear sense of urgency among the various stakeholders around the need to harmonize CHW programs. Another important facilitator included the use of standardized training through the “Training Institute,” which provided training for the government-backed CHWs. The Training Institute was helpful for future changes in the professional development of CHWs and if they would want to integrate more services or other CHW programs into the government-backed CHW role. Other facilitators included the introduction of a governmental pay system (including government-backed CHWs, funded entirely or partly by donors), increased supervision of CHWs, high community satisfaction with the program, and engaging community leaders.

#### Mozambique: Barriers to harmonization

A major barrier was the focus on provincial planning, which created discrepancies among services rendered. There were few staff working at the national scale. Another barrier was the diversity in donor funding by geographical regions, with different programs, monitoring and evaluation indicators, and CHW incentives. Despite having a national CHW program, there were still multiple types of non-MOH CHWs. HIV/AIDS “activists,” for instance, were involved in health advocacy, counselling and testing, home-based care, and ART adherence support. Distribution across the country of various CHW programs was haphazard. Other barriers included the lack of career prospects for CHWs, lack of formal recognition as government employees of the health system (even though they were paid through the governmental pay system), low incentives, delayed payments, and implementation of CHW programs by external actors, an undeveloped financial sector (e.g., limited availability of bank accounts for CHWs making their payment more difficult), and distrust between CHWs and community members, in addition to a general lack of technical, financial, and human resources.

"*I think from a political point of view there seems to be interest. But there seems to be a divergence between that interest and how people allocate resources to it. Because if people don’t allocate resources to it, then they really fail to commit*."

(Expert observer)

## Discussion

Using multicountry case studies, we found that CHWs supporting HIV programs were in general highly fragmented and poorly integrated into national health systems in Southern Africa. We assessed harmonization across 3 priority areas: coordination, integration, and sustainability of CHW programs. Across 4 countries, there are frequently large existing national CHW programs, generally supported in part by the national government. In addition to large-scale government programs, participants often reported a large range of nongovernmental CHW programs delivering HIV services, either alone or in combination with other services. The coexistence of multiple stakeholders supporting CHWs, while generally welcomed (e.g., as an initial emergency response to the HIV epidemic), has often led to multiple parallel systems and fragmentation of services, and could be impeding the potential bigger impact of CHW programs. Frequently mentioned challenges included a lack of oversight and accountability at multiple administrative levels, poor support of CHWs and CHW attrition, and misalignment with a community’s health needs. To our knowledge, this study is the first to develop multicountry case studies to inform decision-making around harmonization of CHW programs for HIV in highly affected countries.

A wide range of facilitating and impedimentary factors were identified to guide policy recommendations and inform decision-making on harmonization of HIV community programs. To make our study’s findings as generalizable as possible to other settings, we extracted overarching mediators of harmonization. Although the countries we assessed are different in many aspects, we found that a large number of mediators were consistent across settings. Key facilitators of harmonization across the study’s countries included (i) a large existing national CHW program and recognition of non-governmental CHW programs, (ii) the use of common incentives and training processes for CHWs, (iii) the existence of an organizational structure dedicated to community health initiatives, and (iv) the involvement of community leaders in decision-making. Other important facilitators of harmonization included the use of local health facilities and political support at all levels of the government. Key barriers to harmonization included (i) a wide range of stakeholders with various guidelines and timelines, (ii) lack of equivalence between training programs of various CHWs, and (iii) lack of ownership and accountability of non-governmental CHW programs, in addition to limited financial and human resources across programs. Future research could explore and test the applicability of each of the mediators identified in our study in other settings. For instance, use of ward-based outreach teams was an important facilitator of harmonization in South Africa [42], but it remains unclear to what extent this reflects an opportunity to achieve harmonization in other countries. Swaziland, for instance, is geographically nested within South Africa and shares many of the health system and contextual characteristics with neighboring KwaZulu Natal province in South Africa. Another important facilitator of harmonization in South Africa that may be of interest to decision-makers in other countries was the use of “clinic committees,” which engaged community leaders and helped create links between communities and health facilities. Conversely, respondents mentioned that Swaziland had integrated specific CHW programs, such as the Red Cross volunteers, shifting from a model of volunteerism to monthly stipends [43]. These experiences may provide important lessons and opportunities for other settings. In Box 2, we show 5 policy recommendations to help achieve harmonization of CHW programs, based on the overarching findings from our 4 country case studies.

**Box 2. Policy recommendations for the harmonization of CHW programs**

1. *Further develop large existing national program and recognize non-MOH CHW programs for HIV*
  - Standardize training/services through the government
  - Employ compatible training levels, career opportunities, and incentives, both provincially and nationally
  - Integrate specific NGO programs into national system in a piecemeal fashion
2. *Place community initiatives under same organizational entity and provide adequate resources to support CHW-led services*
  - Create a clear path of integration for CHWs into a single ministry or large private provider
  - Create a national coordinating agency with sufficient human, technical, and financial resources
  - Hire CHWs and provide better incentives and career prospects to retain existing ones
  - Increase and standardize supervision to oversee CHW programs
3. *Commit stakeholders to meetings and platforms created to coordinate CHW HIV services*
  - Coordinate initial funding, planning, and implementation versus “after the fact” once partners are installed
  - Commit to regular HIV technical working groups
  - Increase number of meetings and/or make attendance mandatory
  - Identify champions to spearhead harmonized programming at different levels
4. *Further engage local health facilities*
  - Make CHW-led HIV services more accountable to local health facilities
  - Develop links with health facilities such as through ward-based outreach teams
  - Provide clinics with adequate capacity to handle CHWs and coordinate between stakeholders
5. *Provide political stability and continuity for community health initiatives involved with HIV services*
  - Include community leaders in harmonization to navigate community politics and different chiefdoms
  - Select clinic or community committees that are representative of the community
  - Utilize window of opportunity with political support in favor of harmonization of CHW programs for HIV
  - Engage HIV advocacy groups, communication offices, and media to sustain momentum

Although this study identified many benefits of harmonizing CHW programs for HIV, respondents also brought up disadvantages to harmonization. Indeed, greater central control of CHWs may not necessarily be positive. Integrating CHWs entirely into a single national health system or large private provider, for instance, could reduce flexibility (e.g., in recruiting community members) or reduce trust by community members in CHWs if they do not trust services provided by the government or other large institution [44]. Although most participants largely appeared in favor, some expressed concerns around increased harmonization. Participants raised questions about how financial and technical harmonization of CHW programs would work in practice. The government’s capacity to provide high quality supervision and management may be limited, and quality of services may deteriorate if CHWs are overburdened with tasks. In Mozambique, the multitude of existing CHW responsibilities reduced their ability to complete additional tasks. Some participants also questioned how to continue tailoring HIV services to community needs, as opposed to only what the government might (mis)understand as being their need, and suggested that such a process might take a long time. Some respondents also voiced concerns that older CHWs may lose their positions if they do not fit new criteria in a harmonized system; and that harmonization of incentives could redefine the scope of work expected of CHWs, thereby changing the rules of engagement for CHWs who were recruited as volunteers. Large national CHW programs are increasingly moving away from volunteerism as a reliable motivator for CHWs to deliver substantial and measurable results long-term. Long-term planning among development partners, such as around the need to exit or transition to a larger provider when program funding ends, may also limit more aspirational planning. The “accompaniment approach,” for instance, suggests to support CHW programs for HIV until countries have the capacity to sustain service delivery [45]. Overall, however, participants saw more advantages than disadvantages to harmonization, such as increased impact of CHW programs, and more long-term solutions to community health issues. Stakeholders were generally supportive of a more harmonized approach to implementing CHW programs.

Finally, in addition to the trade-offs associated with harmonization, we note the importance of the broader health system and context supporting CHW programs. As CHW programs are harmonized and scaled up in settings with scarce resources [33,46,47], a critical question is whether the benefits of these investments will be reaped without concurrent effort to strengthen the broader health system. Integration depends on the health system’s capacity to absorb the program—financially and structurally. CHW programs, for instance, may need to be coordinated with local clinics and teaching facilities that have adequate financial and human resources (e.g., nurses, physicians, teachers) to coordinate CHW programs operating in their catchment area. Similarly, the broader context, including “demographic, economic, political, legal, ecological, sociocultural…and technological factors”, can play a critical role in enabling or hindering the adoption of health sector innovations [34]. The donor environment, such as fiduciary requirements imposed on donor agencies by governing structures, influences the health system but extends beyond it.

### Limitations

This study has a number of limitations. First, our findings are based on case studies which were purposively selected based on the epidemiological context and existence of CHW programs. We were also unable to interview many more relevant decision-makers. However, we generally reached saturation through data triangulation and interviewing a large number of key stakeholders who were well informed. Second, we interviewed stakeholders directly involved in management, support structures, or funding of CHW programs. Our intention was to assess the views of policy-makers and program implementers who make decisions regarding CHW programs and are currently at the forefront of planning and engaging with the expansion of CHWs. Future research should include the bottom-up perspectives from CHW clients, CHWs, and community members. The views of CHW clients are of particular interest, as the end goal of health systems reform is to improve health outcomes as opposed to programs or processes. CHWs are at the forefront of delivery and are able to assess whether the identified issues and mediators of harmonization are feasible. Third, although our results are similar across countries, the findings are country-specific. For instance, it is possible that facilitators to harmonization in one country are barriers in another. While we caution against generalizing to other settings, our findings are likely informative for decision-makers involved with CHW programs, particularly in the context of a large, generalized HIV epidemic, complex health systems, and multiple donors. Fourth, the concepts of CHWs and harmonization are multifaceted and can be interpreted differently by different people. Our interviewers, however, began by asking study participants how they defined CHWs and asked specific questions on clearly defined components of harmonization. Fifth, harmonization is in many cases a challenging and gradual process. In settings with decentralized government, for instance, minor, incremental steps may be required to achieve full harmonization of CHW programs for HIV. “Partial” integration may mean the cadres are not necessarily owned by the MOH, but by community groups, NGOs, or private providers and simply more strongly aligned to national systems. Conversely, countries with a stronger central government, fleeting political will, or a substantial existing national CHW program may be able to achieve effective harmonization quickly. The government of Rwanda, for instance, coordinated salary support to CHWs with an NGO [48], and Brazil and Ethiopia placed their CHWs entirely into an existing civil service structure [16], which considerably facilitated harmonization of their community programs. Finally, we note that harmonization is comprised of multiple interlinked components. There is significant overlap of topics across areas of harmonization and elements of the analytic framework. The perception of a CHW program’s effectiveness among community members and policy-makers, for instance, is important for both integration and sustainability of CHW programs [49,50]. Both coordination and integration into the wider health system are also oft-cited facilitators of sustainability (e.g., for their contribution to be sustained, CHW programs may need to be integrated into the wider health system).

### Conclusion

Multiple disparate CHW programs for HIV exist in 4 Southern African countries. The lack of coordination between CHW programs and the lack of integration of CHW programs into larger health systems may have impeded the full realization of the potential impact of CHWs in HIV. To strengthen and sustain the role of CHWs in HIV service delivery, decision-makers in HIV-endemic settings should take the following actions: further develop government CHW programs; officially recognize nongovernmental CHW programs; standardize CHW training, incentives, and services; provide a dedicated organizational structure for community health initiatives; involve the community in decision-making; and utilize a favorable political window of opportunity for increased harmonization. Adequate and long-term resources are urgently needed to support a more harmonized approach towards CHW programs for HIV.

## Supporting information

S1 COREQ Checklist COREQ 32-item checklist. (DOCX)

S1 Text Review and framework. (DOCX)

S2 Text Additional country context and results. (DOCX)

S1 Box Priority areas for harmonization. (DOCX)

S1 Fig Conceptual framework for analyzing the harmonization of CHW programs. (DOCX)

S1 Table Country data analysis sheet (sample). (DOCX)

S1 Data Primary coded data. (XLSX)

## Abbreviations

APE: Agente Polivalente Elementare
ART: antiretroviral therapy
CHW: community health worker
DHMT: District Health Management Team
DOH: Department of Health
GHWA: Global Health Workforce Alliance
HRH: human resources for health
MOH: Ministry of Health
MOU: memorandum of understanding
NGO: nongovernmental organization
RHM: rural health motivator
VHW: village health worker
