# Supplementary material for: Harmonization of community health worker programs for HIV: A four-country qualitative study in Southern Africa
Source: PLoS Med. 2017 Aug 8;14(8):e1002374. doi: 10.1371/journal.pmed.1002374 (PMC5549708; doi:10.1371/journal.pmed.1002374)
Supplement: S1 COREQ Checklist — (DOCX) [file pmed.1002374.s001.docx]

## S1 Checklist: COREQ 32-item checklist

| **No** | **Item** | **Guide questions/description** |
| --- | --- | --- |
| **Domain 1: Research team and reflexivity** | | |
| Personal Characteristics | | |
| 1. | Interviewer/facilitator | KA and NA conducted all interviews in Lesotho, Mozambique, South Africa and Swaziland, with the exception of Mozambique where Dr. Isabel Cambe (National Institute of Health of Mozambique) conducted additional interviews under the guidance of the study team. |
| 2. | Credentials | JWDN (MD MPH), HGD (BA), KA (MPH), NS (MA MPH), CB (ScD), RG (MD MPH), PG (MBChB MPH), MV (MSc), TB (MD ScD MSc) TJB (PhD), IC (MD) |
| 3. | Occupation | JWDN (Doctoral Candidate in Global Health), HGD (Undergraduate Student), KA (Doctoral Candidate in Global Health), NS (Research Assistant), CB (Research Associate), RG (Clinical Resident), PG (Doctoral Candidate in Global Health), MV (Research Associate), TB (Professor in Global Health), TJB (Senior Lecturer on Global Health Policy and Director of International Health Systems Program), IC (Researcher National Institute of Health of Mozambique) |
| 4. | Gender | All three interviewers were female. |
| 5. | Experience and training | TB, TJB, KA, and IC had extensive experience with conducting in-depth interviews for qualitative research studies. HGD was an undergraduate student, NA a masters student, and JWDN, CB, and PG were doctoral candidates, with training in qualitative research. |
| Relationship with participants | | |
| 6. | Relationship established | None. |
| 7. | Participant knowledge of the interviewer | The participants knew that the researchers were from a university. The context of the interviews and the project overall was informally introduced when interviewers arrived at the interview, and formally as part of the informed consent process. |
| 8. | Interviewer characteristics | The participants knew that the researchers were interested in qualitative research as it pertains to the harmonization of CHW programs for HIV in Southern Africa. |
| **Domain 2: Study design** | | |
| Theoretical framework | | |
| 9. | Methodological orientation and Theory | Multi-country comparative case study analysis using a well-grounded analytical framework introduced by Atun et al. in 2009. |
| Participant selection | | |
| 10. | Sampling | Purposive sampling |
| 11. | Method of approach | Participants were approached by email or phone, based on a preliminary list of 20-25 key stakeholders in each country, made aware of the study, and invited to participate in the study by research staff. |
| 12. | Sample size | 60 |
| 13. | Non-participation | Non-participation was low, at on average 2 participants per country. The most common reasons were being unreachable after several attempts were made and unavailability at the time of our in-country visit (due to either a conference, staff meeting, or senior management retreat). |
| Setting | | |
| 14. | Setting of data collection | Private office or by video conference, with the exception of one interview which was conducted in a public space due to lack of office availability. |
| 15. | Presence of non-participants | No |
| 16. | Description of sample | See Table 1. |
| Data collection | | |
| 17. | Interview guide | An interview guide was drafted based on an extensive narrative review prior to conducting the study and further revised based on comments from expert observers with expertise in qualitative research. |
| 18. | Repeat interviews | None. |
| 19. | Audio/visual recording | Interviews were audio-recorded. |
| 20. | Field notes | Yes |
| 21. | Duration | 30-60 minutes |
| 22. | Data saturation | No |
| 23. | Transcripts returned | No |
| **Domain 3: Analysis and findings** | | |
| Data analysis | | |
| 24. | Number of data coders | Two |
| 25. | Description of the coding tree | A coding tree was not developed. |
| 26. | Derivation of themes | Themes were identified in advance. |
| 27. | Software | No |
| 28. | Participant checking | No |
| Reporting | | |
| 29. | Quotations presented | Yes |
| 30. | Data and findings consistent | Quotations used to illustrate findings. |
| 31. | Clarity of major themes | Yes |
| 32. | Clarity of minor themes | Diverse cases noted and minor themes presented. |
