# Supplementary material for: Harmonization of community health worker programs for HIV: A four-country qualitative study in Southern Africa
Source: PLoS Med. 2017 Aug 8;14(8):e1002374. doi: 10.1371/journal.pmed.1002374 (PMC5549708; doi:10.1371/journal.pmed.1002374)
Supplement: S1 Text — (DOCX) [file pmed.1002374.s002.docx]

## S1 Text: Review and framework

*Narrative review*

We conducted multiple rounds of literature searches in PubMed and Google Scholar using the following English search terms: “harmonization”, “coordination”, “integration”, “sustainability”, “fragmentation”, and “community health worker”. We first arbitrarily selected and reviewed full-text versions of several articles from diverse fields with fragmentation in the title to assess the range of definitions and conceptions of the terms. We then reviewed the first 50 titles of articles which included our search terms anywhere in the text. We reviewed full-text versions of all articles whose primary focus was the harmonization of CHWs. In total, we reviewed full-text versions of approximately 50 articles, book chapters, and case studies. We prioritized articles for analysis that focused on harmonization of CHW programs for HIV. In our full-text review, we searched for and noted (i) evidence on definitions, models, and/or frameworks of the three priority areas of harmonization, (ii) theoretical arguments or hypotheses about the effects of CHW program fragmentation and/or harmonization, and (iii) any relevant empirical evidence. When articles referenced other papers for definitions of harmonization or key empirical evidence of harmonization, we then reviewed those articles. Additional details on our narrative review are provided elsewhere [1].

*Conceptual framework*

In our analyses, we rely upon a framework first proposed by Atun et al. in 2010, displayed in S1 Figure [2]. While the analytic framework was first proposed as an approach to systematically consider the integration of health sector activities, expanding the lens to include all three priority areas enables us to better understand why interventions may fail to achieve harmonization. The framework considers the nature of the health problem, the intervention designed to address it, the stakeholders, the health system itself, and the broader context. Each element can be described as it contributes towards the three components of a harmonized approach. The problem must be considered in light of the urgency and scale of the issue, including the social narrative which surrounds it and the socio-economic structure of distribution. More urgent issues may necessitate a more targeted approach, with efforts for integration occurring further down the line. Less complex and better known interventions are easier to duplicate and likely to be more amenable to integration than newer or more complicated interventions which must be customized to specific target groups (i.e., HIV services are less straightforward than, for instance, childhood immunizations—an easily identifiable target group and schedule). The perceptions and relative power of the various stakeholders is a critical question in the path to integration. The presence of advocates can be a key determinant. Integration further depends on the broader health system’s capacity to absorb the program—financially and structurally. Finally, the broader context, including the “demographic, economic, political, legal, ecological, sociocultural...and technological factors”, can play a critical role in enabling or hindering the adoption of health sector innovations [2].

**References**

1. De Neve J-W, Boudreaux C, Gill R, Geldsetzer P, Vaikath M, Bärnighausen T, et al. Harmonizing community-based health worker programs for HIV: a narrative review and analytic framework. Human Resources for Health. 2017. doi: 10.1186/s12960-017-0219-y.

2. Atun R, de Jongh T, Secci F, Ohiri K, Adeyi O. Integration of targeted health interventions into health systems: a conceptual framework for analysis. Health Policy and Planning. 2010;25(2):104-11. doi: 10.1093/heapol/czp055.
