# Supplementary material for: Harmonization of community health worker programs for HIV: A four-country qualitative study in Southern Africa
Source: PLoS Med. 2017 Aug 8;14(8):e1002374. doi: 10.1371/journal.pmed.1002374 (PMC5549708; doi:10.1371/journal.pmed.1002374)
Supplement: S2 Text — (DOCX) [file pmed.1002374.s003.docx]

## S2 Text: Additional country context and results

Although 60 interviews is relatively large (saturation is quickly reached because only a few decision-makers are really well enough informed about harmonization processes), we extensively engaged with country-specific experts, literature, and other relevant records in the following three ways. First, we conducted an extensive narrative review prior to conducting the study to search for (i) definitions, models, and/or frameworks of harmonization, (ii) theoretical arguments or hypotheses about the effects of CHW program fragmentation, and (iii) empirical evidence. This narrative review informed our conceptual framework and the design of our semi-structured interview guides, and included many country-specific reports and empirical evidence from our target countries. Second, to increase the credibility and validity of our results, we further reviewed country contexts prior to visiting each country and triangulated data from our interviews with published literature, country reports, national health plans, and policies. Finally, in each country, we sought feedback on the study from national experts.

### Swaziland

Swaziland has a long-standing national CHW program, the Rural Health Motivators (RHMs), with currently over 5,000 CHWs [1]. The broad mandate of the RHM program is to provide health information, education and communication to its population with the aim of changing people’s attitude and behavior on a range of health issues. In addition, numerous donors and NGOs have created a number of smaller CHW programs that deliver HIV services, such as the “expert clients” and “mothers2mothers”.

While the RHM program aims to be more holistic and all-encompassing than HIV alone, a lot of parallel efforts have been created to address HIV specifically. Although some of these efforts may have been a result of the emergency response to address the HIV epidemic, interviewees mentioned that a variety of services were being provided to deal with HIV, which has proven to be difficult to coordinate. A few programs also aim to coordinate HIV with other health issues, such as TB and maternal, newborn and child health (MNCH), given the intersections of different health issues and prevalence within communities already dealing with HIV; although this appears to have added complexity to any rendering of services for HIV.

Different CHW programs have different requirements. Interviewees mentioned the need to coordinate the education level between them. Many of the older RHMs have not completed secondary education and any new educational requirements would mean they have to be “grandfathered in” if they were to continue being RHMs. Younger CHWs, on the other hand, would have the technical capacity but increasingly want to move outside of rural areas to seek better employment opportunities. If younger CHWs could not be recruited and retained within the program, then there are concerns for improving technical capacity in the long run as many seek more gainful employment elsewhere.

While there are some parallel chains (some reported getting all their supplies from facilities, while others give their CHWs their own kits), many of the CHW programs are working on similar timelines and with similar materials when they do use supplies. Finally, many CHWs are also trained in siSwati whereas other healthcare workers are trained in English—these language differences appear to further impact forms and reporting.

*Swaziland: additional quotes*

*“The ministry … they have also got limited resources. That’s my understanding that they can’t absorb everything. But eventually in the long-run yes, they have to absorb it. But maybe not at once.”* (Expert observer)

*"Being young, they’re not going to be staying in this area, this is a rural set up. People are going into towns looking for potential jobs. So we’re not going to have a stable or a set number of RHMs. The ones that we saw so far …. They cannot read or write.”* (Expert observer)

*"So when we do our coordination if we can start using Ministry of Tinkhundla and community engagement guidelines we can be able to collaborate and be able to coordinate and implement these activities together."* (Expert observer)

*“The one thing I think about Swaziland and the health sector is that there is a lot of political will to make sure the health sector succeed. Whether there is political support directly to the community health cadre, I can’t exactly say but I don’t foresee it being a challenge because all other community initiatives have always been supported politically, but there is even I think political commitment to wanting more outreach, a lot more health initiatives being done within the community."* (Government official)

*“We have developed a package of health services that we feel people at a community level can give to their communities. So because of this document we want to start encouraging a sort of standard across the community healthcare workers….”* (Government official)

*“People who want to go to the community should report to the ministry of health and work with the RHM program because the RHMs know where there are more health providers and where there are gaps.”* (Government official)

*"There is competition and conflict between the very same programmers that are delivering services...”* (Expert observer)

*“It [harmonization] will raise an expectation…From the people who own the [CHW] program, who are also called owners of the program. That now the Ministry wants to control us.”* (Expert observer)

### South Africa

The total number of “community service health professionals” in South Africa was estimated at 44,000 in 2014 [2]. CHWs have increasingly become key players in public health care, particularly for provision of ARV treatment services [3, 4]. Despite broad support for the concept of primary healthcare, a national CHW program has been slow to develop [4].

In addition to other factors reported in the main text, participants voiced concern over interagency coordination, and the lack of coordination between different government departments themselves to increase harmonization of CHW programs for HIV.

*South Africa: additional quotes*

*"There are still those that are employed by NGOs and we don’t have maybe as strong a control, I mean mechanism, in terms of supervising those CHWs….."* (Government official)

*"… Some of the districts have not budgeted for the training of CHWs."* (Donor)

*"Post-1994, we closed down a number of nursing colleges. As well as colleges of education, now we are sitting with problems of we don’t have enough resources to do these two sectors... So you set up a clinic that you know you will struggle to staff that clinic, ok. So when patients die, when mothers and babies die, it’s a reflection of the system, yes."* (Government official)

*"The community health worker has as structured training, they also got through a focused assessment that is actually checking their skills competency, whether they are able to provide the basic primary healthcare at household level or not….. So it is a means of taking them through a career path if they want to continue, in this career path, to be a nurse they can do that."* (Expert observer)

*"We require they [DONOR/NGO] use the same path even if they do their own training, but when they do their own training we want them to link up with our regional training center...and ensure that they use the manual of the department of health, or it is not the department of health but it is an accredited manual at our regional training center must do the curriculum training."* (Government official)

*"The NGOs … work via health facilities that are there, they introduce themselves by the health facilities and it is based on what health facilities refer to them and say go and visit house 101 and they will visit that house and support that household."* (Government official)

*“… The CHW program and the ward-based outreach team should be...linked to the clinic facility…”.* (Expert observer)

*“Because of uneven development of the health systems in the 9 provinces, I think that when the actual [CHW] program will be rolled out, it will be very uneven, it will be very different in each provinces.”* (Donor)

### Lesotho

A recent assessment estimated the number of in Lesotho at 7,103 [5]. Their role in HIV service delivery include a wide range of community services, including door-to-door testing campaigns, pre- and post-test and ART adherence counselling, monitoring ART adherence, and the supply of ARTs at the community level [6].

*Lesotho: additional quotes*

*"But the challenge comes when it is a partner-based community health worker, because now they are not integrated into the health center itself. So you’d be, the partner will be going to the community that they are dealing with and be providing information, in a real life situation it would be better, if I am a partner, I go through the health center."* (Expert observer)

*“You have CHWs, VHWs, peer educators, there are quite a number of cadres that you can pick from.”* (Expert observer)

*"… You know most projects are erratic and they come and go and cycles, so it creates these waves of, we work for this and if gone, who is there in terms of it. ...when the funding from [DONOR] was ended, they were getting another one but still there was that challenge in who is going to take these VHWs or CHWs?"* (Expert observer)

*"We need somebody who is much stronger and who has more influence to coordinate, because partners, I think, that will sort of not be organized well if we are to say we should organize ourselves as partners."* (Expert observer)

*"People don’t want to test in the same communities as the community health worker that they know of..."* (Expert observer)

*"This country is so small, we can reach any part of the country most 8 hours, no matter how bad the terrain. … You have a population of 2.1 million. Anything that you do, the impact is massive, you know. You know where your population pockets are.”* (Expert observer)

*“The role of government is really to create a platform for different stakeholders to come together, developing training materials, do the necessary changes, so when trainings are conducted, everything is uniform.”* (Donor)

*"So with different players being around, I don’t think the government can absorb all the community health workers, that’s why they want to streamline and standardize things only what is manageable within their means."* (Expert observer)

*"But that is one aspect which I think we have failed, we have not been able to, the village health workers have been trained but following them up has not been something that is consistent or done on a periodic basis."* (Expert observer)

*“… Right now the issue that somebody will be paying them while other, those that are working for government probably are not paid or paid little, little amount of money. It brings dissatisfaction.”* (Expert observer)

*"’Test and start’, whether we are even thinking of advocating for circumcision before the initiation school, so again now you need men. But if VHWs are women, how do you approach men on issues such as that?"* (Expert observer)

### Mozambique

Large scale CHW programs in Mozambique were introduced in the mid-1970s. The national CHWs are known as “Agentes Polivalentes Elementares” (APEs) (“essential [or elementary] multi-purpose agents”) [7]. Their role focuses on providing broad primary healthcare services to remote rural communities. The program was discontinued in 1989, until a revitalized APE national program was rolled out in 2010 to increase the coverage and quality of primary healthcare. As of December 2013, there were 2270 APEs trained, covering 12% of the population of Mozambique [8].

National CHWs in Mozambique are largely supported by a multitude of different funders, such as the World Bank, World Vision, USAID, and Save the Children [9]. The multitude of different funding streams and the contradictory accountability systems faced by managers in the health system are a major factor in the harmonization of the national CHW program [10-12]. A key question in the literature is whether the renewed national CHW program is more sustainable [9, 13]. Boon 2015 points to two phases in the scale up of CHWs: (i) a ‘launching/revitalizing phase’, when there is a need for funds, coverage, and different funding mechanisms, which is followed by (ii) a ‘stabilizing phase’, when there is a need for more alignment of planning periods among donors and partners, consistency of support, and transition to local ownership and resources [14]. Our study is particularly timely as countries towards increased harmonization during the stabilization phase.

*Mozambique: additional quotes*

*"… we're just all working for the same purpose, one pay this something [while the] other is doing something else"* (Donor)

*"Expansion of CHW to other districts beginning when it was revitalized, were only 8 districts now we are in 137"* (Government official)

*“It is my understanding that it is the Ministry of Health. I think before different partners were working on projects separately but about 3 years ago the ministry and donors tried to bring it back under the ministry’s supervision. So the planning is basically coordinated within the Ministry...But it seemed as though that the province should be managed by the Ministry of Health. Although they are different types of support, and I know of at least 2 NGOs that also support the province ….”* (Expert observer)

*“Donor-funded partners don’t come to the Department [of Health], they just go to the community. We discovered that when they are implementing, when you visit a household they are indicating that somebody form this organization was here, then we have to track them down, invite them to our partnership meetings, and explain to them how we are working ...” (Government official)*

*“… The CHW wants to progress, it does not want to be a lifetime CHW.”* (Government official)

*“We wanted all CHWs had bank accounts, but [this] took a long time…”* (Donor)

**References for appendix**

1. East, Central, and Southern African Health Community (ECSA-HC). Task shifting in Swaziland: A case study. Futures Group Health Policy Initiative. 2010.

2. National Department of Health of South Africa. Social Sector Cluster Media Briefing. 2014.

3. van Ginneken N, Lewin S, Berridge V. The emergence of community health worker programmes in the late apartheid era in South Africa: An historical analysis. Social Science & Medicine. 2010;71(6):1110-8. doi: 10.1016/j.socscimed.2010.06.009.

4. Schneider H, Hlophe H, van Rensburg D. Community health workers and the response to HIV/AIDS in South Africa: tensions and prospects. Health Policy and Planning. 2008;23(3):179-87. doi: 10.1093/heapol/czn006.

5. Khabo M, Matinhure S, Maema M. Lesotho village health worker's profiling and mapping exercice report. Human Resources Alliance for Africa. 2013.

6. Mwai GW, Mburu G, Torpey K, Frost P, Ford N, Seeley J. Role and outcomes of community health workers in HIV care in sub-Saharan Africa: a systematic review. Journal of the International AIDS Society. 2013;16:18586. doi: 10.7448/IAS.16.1.18586. PubMed PMID: 24029015; PubMed Central PMCID: PMC3772323.

7. Lindelow M, Ward P, le Guillouzic-Zorzi N. Primary Health Care in Mozambique. Service Delivery in a Complex Hierarchy. World Bank Africa Region Human Development Working Paper Series No 69 20032003.

8. Ministerio da Saude. Relatorio Anual das Actividades do Programa de Agentes Polivalentes Elementares (APEs) do Ano 2013. Maputo: Mozambique: Ministerio de Saude; 2014b.

9. Chilundo BGM, Cliff JL, Mariano ARE, Rodríguez DC, George A. Relaunch of the official community health worker programme in Mozambique: is there a sustainable basis for iCCM policy? Health Policy and Planning. 2015;30(suppl 2):ii54-ii64. doi: 10.1093/heapol/czv014.

10. Bärnighausen T, Bloom DE, Humair S. Global Health Governance and Tropical Diseases. In: Jeremy Farrar NW, David Lalloo, Peter Hotez, Thomas Junghanss and Gagandeep Kang, editor. Manson’s Tropical Diseases 23rd Edition: Elsevier; 2014. p. 16-22.e1.

11. Bowser D, Sparkes SP, Mitchell A, Bossert TJ, Barnighausen T, Gedik G, et al. Global Fund investments in human resources for health: innovation and missed opportunities for health systems strengthening. Health Policy and Planning. 2013;29(8):986-97. doi: 10.1093/heapol/czt080.

12. McCoy D, Chand S, Sridhar D. Global health funding: how much, where it comes from and where it goes. Health Policy and Planning. 2009;24(6):407-17. doi: 10.1093/heapol/czp026.

13. Ndima SD, Sidat M, Give C, Ormel H, Kok MC, Taegtmeyer M. Supervision of community health workers in Mozambique: a qualitative study of factors influencing motivation and programme implementation. Human Resources for Health. 2015;13(1). doi: 10.1186/s12960-015-0063-x.

14. Boon A. Importance of Donor Coordination in Mixed Health Financing in the SDG Era. Financing for Community Health Workers and Health Systems at Scale in sub-Saharan Africa. 2015.
