# Supplementary material for: Harmonization of community health worker programs for HIV: A four-country qualitative study in Southern Africa
Source: PLoS Med. 2017 Aug 8;14(8):e1002374. doi: 10.1371/journal.pmed.1002374 (PMC5549708; doi:10.1371/journal.pmed.1002374)
Supplement: S1 Box — (DOCX) [file pmed.1002374.s004.docx]

# S1 Box: Priority areas for harmonization

*Coordination:* Any activity or set of activities undertaken to ensure that foreign inputs into the health sector enable the health system to function more effectively and in accordance with local priorities over time [1]. Among CHW programs, coordination efforts seek to reduce duplication, fragmentation, confusion created by competing models, and overlap of responsibilities of differently trained CHW in the same geographic areas [2, 3].

*Integration:* The absorption of CHW programs into existing networks of larger health systems, primarily the Ministries of Health or large private providers (NGO or commercial). Integration is defined as “the extent, pattern, and rate of adoption and eventual assimilation of health interventions into each of the critical functions of a health system” [2].

*Sustainability:* “The continued use of program components and activities for the continued achievement of desirable program and population outcomes” [4]. Sustainability is a key element of CHW-led HIV services which are transitioning out of vertically funded sources (e.g., PEPFAR, Global Fund).

**References**

1. Buse K, Walt G. Aid coordination for health sector reform: a conceptual framework for analysis and assessment. Health policy. 1996;38(3):173-87. PubMed PMID: 10162420.

2. Atun R, de Jongh T, Secci F, Ohiri K, Adeyi O. Integration of targeted health interventions into health systems: a conceptual framework for analysis. Health Policy and Planning. 2010;25(2):104-11. doi: 10.1093/heapol/czp055.

3. Mogedal S, Wynd S, Afzal MM. Community Health Workers and Universal Health Coverage: A Framework for Partners’ Harmonized Support. 2013.

4. Pallas SW, Minhas D, Perez-Escamilla R, Taylor L, Curry L, Bradley EH. Community health workers in low- and middle-income countries: what do we know about scaling up and sustainability? American journal of public health. 2013;103(7):e74-82. doi: 10.2105/AJPH.2012.301102. PubMed PMID: 23678926; PubMed Central PMCID: PMC3682607.
