# Supplementary material for: Harmonization of community health worker programs for HIV: A four-country qualitative study in Southern Africa
Source: PLoS Med. 2017 Aug 8;14(8):e1002374. doi: 10.1371/journal.pmed.1002374 (PMC5549708; doi:10.1371/journal.pmed.1002374)
Supplement: S1 Fig — (DOCX) [file pmed.1002374.s005.docx]

# S1 Figure: Conceptual framework for analyzing the harmonization of CHW programs


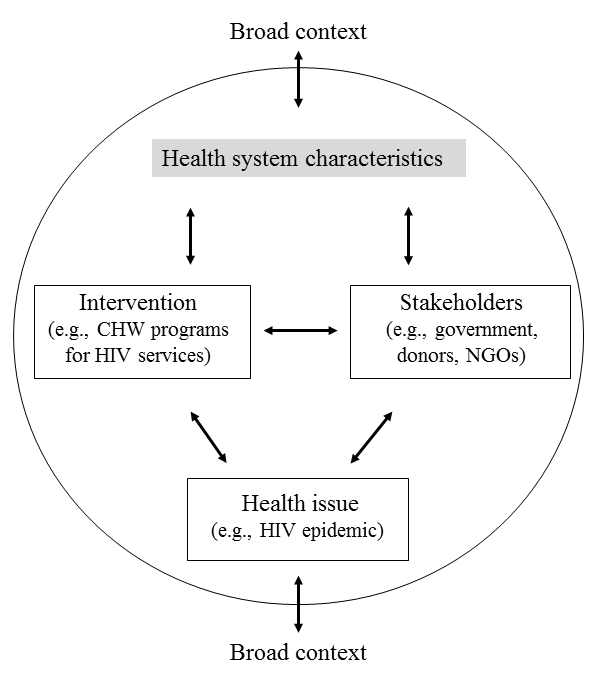


Figure displays the analytic framework, which considers the nature of the health issue of concern, the intervention designed to address it, the stakeholders, the health system, and the broader context (Atun et al., 2010). Each element can be described as it contributes towards harmonizing CHW programs for HIV.
