# Supplementary material for: Harmonization of community health worker programs for HIV: A four-country qualitative study in Southern Africa
Source: PLoS Med. 2017 Aug 8;14(8):e1002374. doi: 10.1371/journal.pmed.1002374 (PMC5549708; doi:10.1371/journal.pmed.1002374)
Supplement: S1 Table — (DOCX) [file pmed.1002374.s006.docx]

# S1 Table: Country data analysis sheet (sample)

*Notes:* Table shows the data analysis sheet (sample), separately for each country, including potential disadvantages to harmonization to assess whether respondents reported concerns about increased coordination, integration, and sustainability of CHW programs for HIV.
